# From Frequencies to Priorities: Interethnic Distribution of Pharmacogenetic Phenotypes as a Basis for Prioritizing Preemptive Testing in Russia’s Multiethnic Population

**DOI:** 10.3390/genes17101275

**Published:** 2026-10-14

**Authors:** Karin B. Mirzaev, Sherzod P. Abdullaev, Svetlana N. Tuchkova, Firuz Kh. Turaev, Lyubov V. Selivanova, Denis S. Fedorinov, Dmitry A. Sychev

**Affiliations:** 1 Petrovsky National Research Center of Surgery, Moscow 119435, Russia; 2 Russian Medical Academy of Continuous Professional Education, Moscow 125993, Russia; 3 Egoryevsk Hospital of the Moscow Region, Egoryevsk 140301, Russia; 4 Moscow Multidisciplinary Clinical Center “Kommunarka”, Moscow 142770, Russia

**Keywords:** pharmacogenetics, CYP2C9, CYP2C19, CYP3A5, SLCO1B1, VKORC1, CYP4F2, ethnic diversity, warfarin, preemptive testing, personalized medicine, health policy prioritization, Russia

## Abstract

Background/Objectives: The frequencies of pharmacogenetically relevant alleles and predicted phenotypes vary substantially between populations, with direct consequences for the expected efficacy and safety of widely prescribed drugs. Russia is one of the most ethnically diverse countries in the world, yet systematic, multi-gene pharmacogenetic data across its ethnic groups, and their implications for targeted implementation of testing, remain limited. This study aimed to characterize the distribution of predicted metabolizer phenotypes for CYP2C9, CYP2C19 and CYP3A5 and of predicted SLCO1B1 transporter function, together with VKORC1 and CYP4F2 genotypes, across 15 ethnic groups of Russia, and to establish the population-level frequency data required for subsequent analysis of regional priorities for pharmacogenetic testing. Methods: A total of 2304 unrelated individuals from 15 ethnic groups were genotyped for CYP2C9*2/*3, CYP2C19*2/*3/*17, CYP3A5*3, SLCO1B1*5, VKORC1 c.-1639G>A, CYP4F2*3, and ABCB1 c.3435C>T. Allele, genotype, and phenotype frequencies were estimated with 95% Wilson confidence intervals. Interethnic differences were assessed using the Fisher exact test (omnibus and pairwise comparisons against the Russian reference group), with the Benjamini–Hochberg false discovery rate correction; composite phenotype categories were further compared using 2 × 2 exact tests. Genotype-based expected warfarin maintenance dose ranges were assigned from CYP2C9 diplotype combined with VKORC1 genotype according to the FDA-approved prescribing information, with CYP4F2*3 carriage considered as a modifying factor. Results: Interethnic variability differed markedly by gene, ranging from minimal for CYP3A5 to pronounced for VKORC1. After correction for multiple comparisons, statistically significant differences from the Russian reference group were identified in up to eight of 14 ethnic groups per gene, affecting CYP2C9-, CYP2C19-, and SLCO1B1-related phenotypes relevant to NSAIDs, clopidogrel, proton pump inhibitors, antidepressants, and statins. The most pronounced pattern was a coordinated shift across CYP2C9 and CYP2C19 in Buryats and Nanais consistent with previously reported East Asian population patterns, alongside an isolated, population-specific extreme in Lezgins, who showed the highest CYP2C9 intermediate/poor metabolizer frequency of all 15 groups. These differences translated into markedly divergent distributions of genotype-based expected warfarin dose ranges, with the lowest range (0.5–2 mg/day) expected in 21.1% of Lezgins, compared with 8.8% overall, and the 3–4 mg/day range predominating in Buryats and Nanais (69.3% and 73.9%). Conclusions: These findings characterize the pharmacogenetic landscape of Russia’s understudied ethnic diversity and provide the population-level frequency data required as one component of any subsequent assessment of where preemptive pharmacogenetic testing should be prioritized–an assessment that additionally requires data on prescribing patterns, clinical outcomes, cost-effectiveness and healthcare infrastructure. The magnitude of the interethnic differences documented here nonetheless indicates that implementation planning guided by population-specific rather than nationally averaged frequencies merits further evaluation in a multiethnic country.

## 1. Introduction

Interindividual variability in drug response is a principal cause of treatment failure and of adverse drug reactions. A substantial part of this variability is explained by heritable differences in genes encoding drug-metabolizing enzymes and transporter proteins [1]. The implementation of pharmacogenetic testing into clinical practice–selecting a drug and its dose according to a patient’s individual genotype–is recognized as one of the most readily implementable directions of personalized medicine to date, for which formalized clinical guidelines (CPIC, DPWG) have been developed for specific gene–drug pairs. Beyond its clinical relevance, pharmacogenetic testing also has an economic dimension. A systematic review of economic evaluations of personalized therapy for gene–drug pairs from the FDA biomarker list showed that more than half of such evaluations favor testing, and nearly a third represent a cost-saving strategy [2]. For drugs with CPIC guidelines, a similar conclusion was confirmed by a more recent systematic review [3].

The frequencies of clinically significant allelic variants of CYP2C9, CYP2C19, CYP3A5, and SLCO1B1 differ substantially among world populations, as confirmed by studies conducted across different regions. In Latin America, the RIBEF-CEIBA consortium, which pooled data from more than 6000 Indigenous and admixed Ibero-American individuals, showed that CYP2C9 and CYP2C19 allele and phenotype frequencies are directly related to the proportion of Amerindian, European, and African genomic ancestry components in an individual and differ substantially even between neighboring countries of the same region [4,5]. In East and Southeast Asia, the frequency of the reduced-function allele CYP2C19*3 is 2–3 times higher than in individuals of European ancestry, which produces a risk profile for therapeutic failure–for example, with clopidogrel and several antidepressants–that differs fundamentally from that described in Western guidelines [6,7]. In the United States, the issue is viewed from a different angle: the overwhelming majority of genome-wide pharmacogenetic studies have historically been conducted in cohorts of European-ancestry descendants, so that the resulting findings and the dosing algorithms built on them perform worse in African Americans and other ethnic minority groups [8].

Population studies of pharmacogenetic marker distribution are increasingly regarded not merely as a descriptive undertaking but as a tool for prioritizing the implementation of testing. Detailed knowledge of population-level pharmacogenetic marker/phenotype frequencies is a prerequisite for evidence-based healthcare resource planning: such data are a necessary input to subsequent analyses of where preemptive genotyping is likely to yield the greatest clinical benefit and to the definition of priority regions and gene–drug pairs for phased implementation of testing. A comparable approach was applied in characterizing pharmacogenetic biomarkers (CYP2C9, CYP2C19, CYP3A5, VKORC1, SLCO1B1, TPMT) across 18 European countries with, at the time, limited access to such data [9], where the authors not only described the spectrum of allele frequencies but also argued for the use of these data in developing national pharmacogenetic implementation strategies in countries with differing income levels. A similar approach also underlies the current initiative to develop regional pharmacogenetic guidelines for Latin America that account for local allele frequency structure rather than direct extrapolation of the CPIC/DPWG guidelines [10].

Russia is one of the most ethnically diverse countries in the world: the most recent national census recorded more than 190 nationalities, a substantial proportion of which maintain territorially distinct settlement patterns (the republics of the North Caucasus, the Volga region, Siberia, the Far East). Data on clinically significant pharmacogenetic markers available to date remain fragmentary [11] and typically cover a single ethnic group and one or two genes [12,13,14]. Particular attention is warranted for a recent study by Yudin et al. (2025), conducted on a representative nationwide sample of Russians with a methodologically rigorous comparison of risk regions based on CYP2C9, CYP2C19, and CYP2D6 allele frequencies [15]. However, that study did not examine clinically significant metabolic phenotypes broken down by individual ethnic group, which is consistent with the fact that a nationwide sample is not, by design, suited to such a comparison. A systematic characterization of these genes and their clinically significant phenotypes across a broad range of individual ethnic groups in Russia has not previously been undertaken.

The aim of the present study was to characterize the distribution of predicted metabolizer phenotypes for CYP2C9, CYP2C19 and CYP3A5 and of predicted SLCO1B1 transporter function, as well as the VKORC1, CYP4F2, and ABCB1 genotypes, across 15 ethnic groups residing in Russia.

## 2. Materials and Methods

The study was approved by the local ethics committee of the Russian Medical Academy of Continuous Professional Education. Prior to enrollment, written informed consent (in Russian) was obtained from all participants for study participation and for the collection and storage of genetic material. Under the terms of the informed consent, the study results could be used for research purposes without disclosure of personal data.

Study population. The study included 2304 apparently healthy volunteers (men and women) from different ethnic groups residing in the Russian Federation: 136 from the Russian ethnic group, 117 Avars, 121 Dargins, 116 Laks, 127 Kumyks, 123 Lezgins, 203 Kabardians, 196 Balkars, 244 Ossetians, 141 Tatars, 206 Mari, 236 Chuvash, 109 Mordvin-Moksha, 114 Buryats, and 115 Nanais.

All participants provided voluntary informed consent to participate in the study and for the collection and storage of genetic material.

Inclusion/exclusion criteria. Ethnic affiliation was determined by self-identification of participants and their parents. As shown in previous studies, self-identification correlates strongly with microsatellite marker-based determination of ethnic origin [16]. Descendants of interethnic marriages were not included in the study.

### Selection of Pharmacogenetic Markers for the Study

The panel of pharmacogenetic markers was assembled based on two criteria: established clinical significance according to the Clinical Pharmacogenetics Implementation Consortium (CPIC) guidelines and assignment of the variant to the minimal (Tier 1) or extended (Tier 2) diagnostic set under the joint recommendations of the Association for Molecular Pathology (AMP) for the corresponding gene. ABCB1 rs1045642 meets neither criterion and was included as an exploratory marker.

The final panel included variants of CYP2C9 (*2, *3), CYP2C19 (*2, *3, *17), CYP3A5 (*3), SLCO1B1 (*5, rs4149056), VKORC1 (rs9923231), and CYP4F2 (*3), as well as ABCB1 (rs1045642) (Table 1).

To compare the frequencies obtained in the studied Russian ethnic groups with global data, the standardized ClinPGx biogeographic grouping system, comprising nine groups, was used: African/Afro-Caribbean, American, Central/South Asian, East Asian, European, Latino, Near Eastern, Oceanian, and Sub-Saharan African [17]. Comparison of the frequencies of the studied groups with ClinPGx reference biogeographic data was descriptive, as the present study interrogated a defined subset of alleles whereas the ClinPGx reference distributions may incorporate broader allele definitions, which would bias the present data toward a higher normal metabolizer proportion.

Predicted CYP2C9, CYP2C19, and CYP3A5 metabolizer phenotypes and SLCO1B1 functional status were assigned from the diplotype using the official CPIC diplotype–phenotype tables for each gene. Diplotypes for CYP2C9 and CYP2C19 were constructed from the individual SNP genotypes listed in Table 1. As the variants were typed individually and no statistical phasing was performed, individuals heterozygous at two positions are formally ambiguous with respect to haplotype phase; in such cases, the variants were assumed to lie on different haplotypes, consistent with the convention of the CPIC diplotype–phenotype tables. For CYP2C9, phenotype was assigned via the summary activity score (AS) system in accordance with standard algorithms. For CYP2C9 and CYP3A5, phenotypes were classified as Normal, Intermediate and Poor Metabolizer; for SLCO1B1, functional status was classified as Normal, Decreased and Poor Function; for CYP2C19, phenotypes were additionally classified as Rapid and Ultrarapid metabolizer, reflecting the presence of the increased-function allele CYP2C19*17.

The CYP2C9 phenotype and VKORC1 c.-1639G>A (rs9923231) genotype are the two principal genetic determinants of warfarin dose, jointly accounting for approximately one-third of interindividual variability in maintenance dose in individuals of non-African ancestry. In individuals of non-African ancestry, the CYP2C9 + VKORC1 combination is assigned the highest level of evidence (Strong) in the CPIC system for grading the clinical significance of gene–drug pairs [18]. This same combination underlies the table of expected maintenance dose ranges (High, Intermediate, Low) in the FDA-approved warfarin prescribing information [19,20]. The CYP4F2*3 genotype (rs2108622) is assigned a lower level of evidence (Optional) in the CPIC guideline and is treated as a modifying factor: carriage of the *3 allele (c.1297G>A, rs2108622) is recommended to increase the dose by 5–10% within the category already established by CYP2C9/VKORC1 [18,21]; the magnitude of this effect has been confirmed in more recent meta-analyses [22].

Accordingly, CYP4F2*3 carriage is reported alongside the dose categories as a modifying factor rather than as a separate category. Each individual was assigned to one of the three expected maintenance dose ranges (5–7, 3–4 and 0.5–2 mg/day) on the basis of their CYP2C9 diplotype (*1/*1, *1/*2, *1/*3, *2/*2, *2/*3, *3/*3) combined with the VKORC1 c.-1639G>A genotype (GG, GA, AA), as specified in the FDA-approved warfarin prescribing information [19,20]; the metabolizer phenotype was not used for this assignment, since diplotypes sharing a phenotype category can correspond to different expected dose ranges. The distribution of these genotype-based expected dose ranges was analyzed in each of the 15 ethnic groups studied (Section 3.4).

Genotyping. Genotyping was performed on 4 mL of venous blood drawn from the cubital vein using the VACUETTE vacuum blood collection system (Greiner Bio-One, Kremsmünster, Austria) into EDTA tubes. DNA was extracted using the DNA-Extran-1 kit for genomic DNA extraction from whole blood (Syntol LLC, Moscow, Russia).

Carriage of the studied polymorphic gene markers was determined by real-time polymerase chain reaction (real-time PCR) using SNP-Screen reagent kits (Syntol LLC, Moscow, Russia) on a CFX96 Touch Real-Time PCR System (Bio-Rad Laboratories, Inc., Hercules, CA, USA), following the manufacturers’ kit protocols. Each sample was assayed in triplicate, and each run included the three positive controls supplied with the kit together with a no-template control. Genotypes were called automatically by the CFX Manager software version 3.0 (Bio-Rad Laboratories, Inc., Hercules, CA, USA) and additionally verified manually; samples that did not yield a call were re-assayed. Across the 15 ethnic groups, 23,035 of 23,040 genotype calls were obtained (call rate 99.98%); of the five failed calls, four were for SLCO1B1 c.521T>C in the Nanai group, and one was for CYP4F2*3 in the Russian group, and the affected individuals were excluded only from the analyses of the corresponding marker.

Genotyping was not performed by star-allele sequencing, and the panel therefore covers a defined subset of the Tier 1 alleles listed in Table 1: for CYP2C9, *2 and *3 account for the large majority of reduced- and no-function alleles in European and Eurasian populations, whereas the remaining Tier 1 alleles (*5, *6, *8, *11) occur predominantly in populations of African ancestry, as do CYP3A5*6 and *7 relative to the *3 allele assayed here. For SLCO1B1, only c.521T>C (rs4149056) was assayed; this variant underlies the CPIC statin dosing recommendations, but the data do not constitute a comprehensive characterization of the broader CPIC SLCO1B1 allele set, and the functional categories reported here are based on this single variant.

Statistical analysis. Genotype frequencies were assessed for consistency with the Hardy–Weinberg equilibrium using Pearson’s χ^2^ test with one degree of freedom, where the expected count of the rarer homozygote was below five, the exact test.

Allele and genotype frequencies were calculated as the ratio of the number of corresponding alleles (genotypes) to the total number of alleles (observations) analyzed in each ethnic group. Confidence intervals (CIs) for proportions were calculated using the Wilson score interval method at the 95% confidence level [23].

The Fisher exact test was used to compare phenotype distributions among ethnic groups. Pairwise comparisons were then performed between each of the 14 groups and the reference Russian group, as the most numerous ethnic group in Russia; *p*-values were corrected using the Benjamini–Hochberg method, controlling the expected false discovery rate (FDR) at 5% [24].

**Figure 1:**
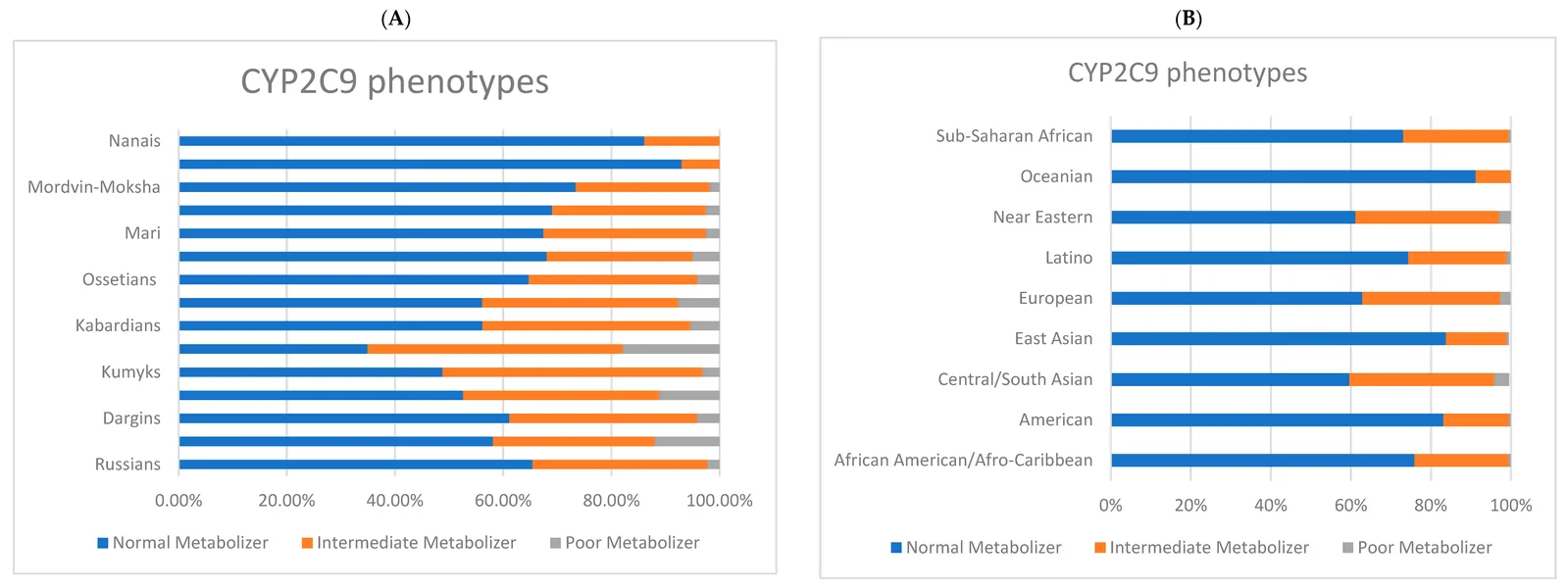

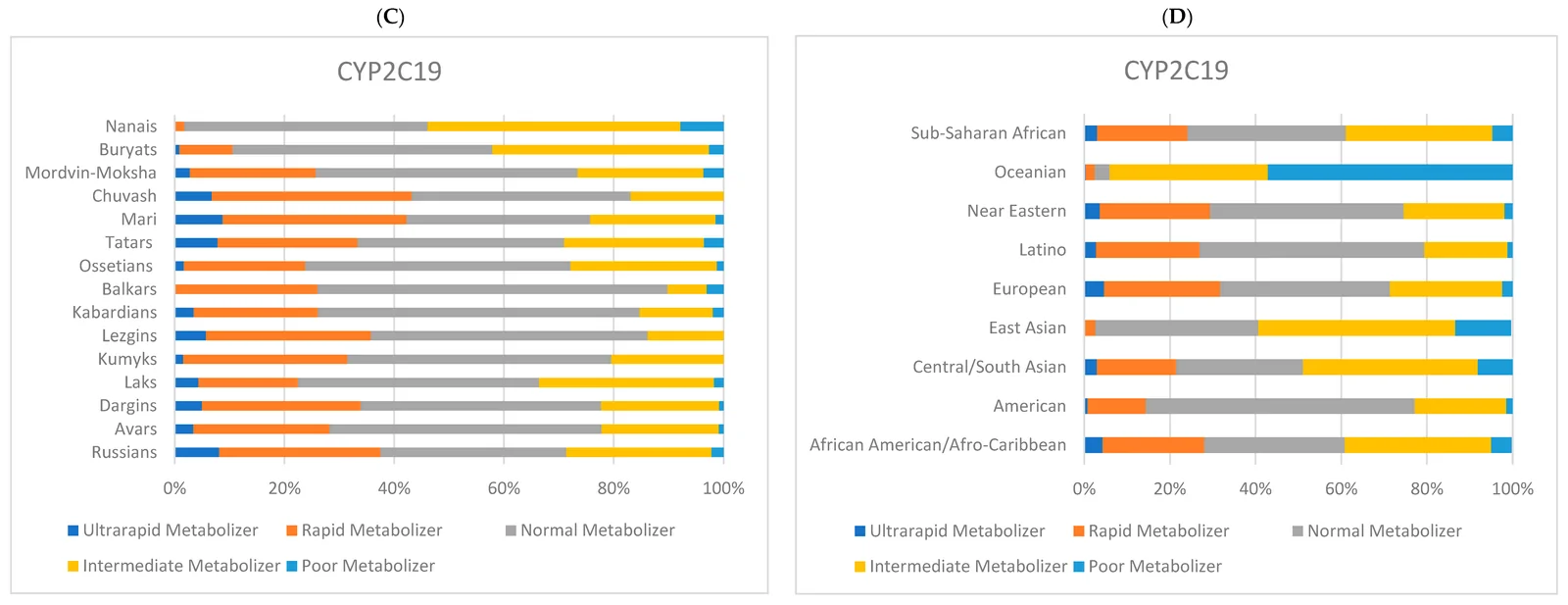

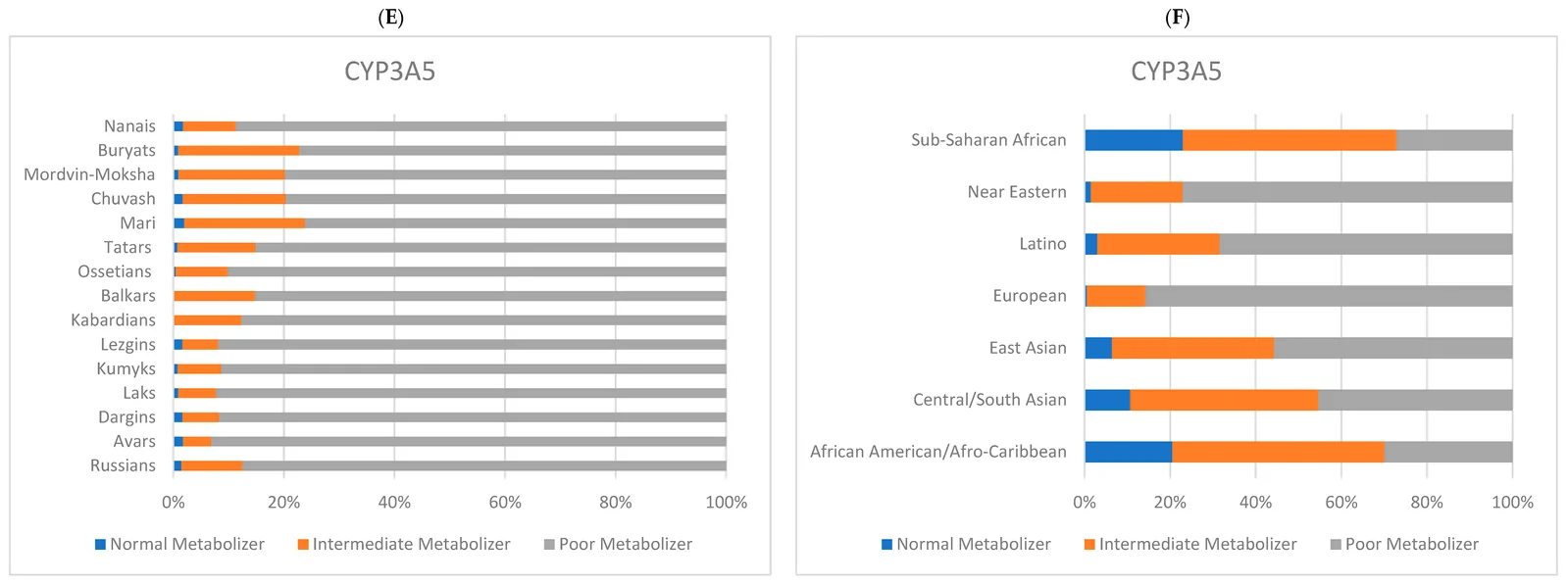

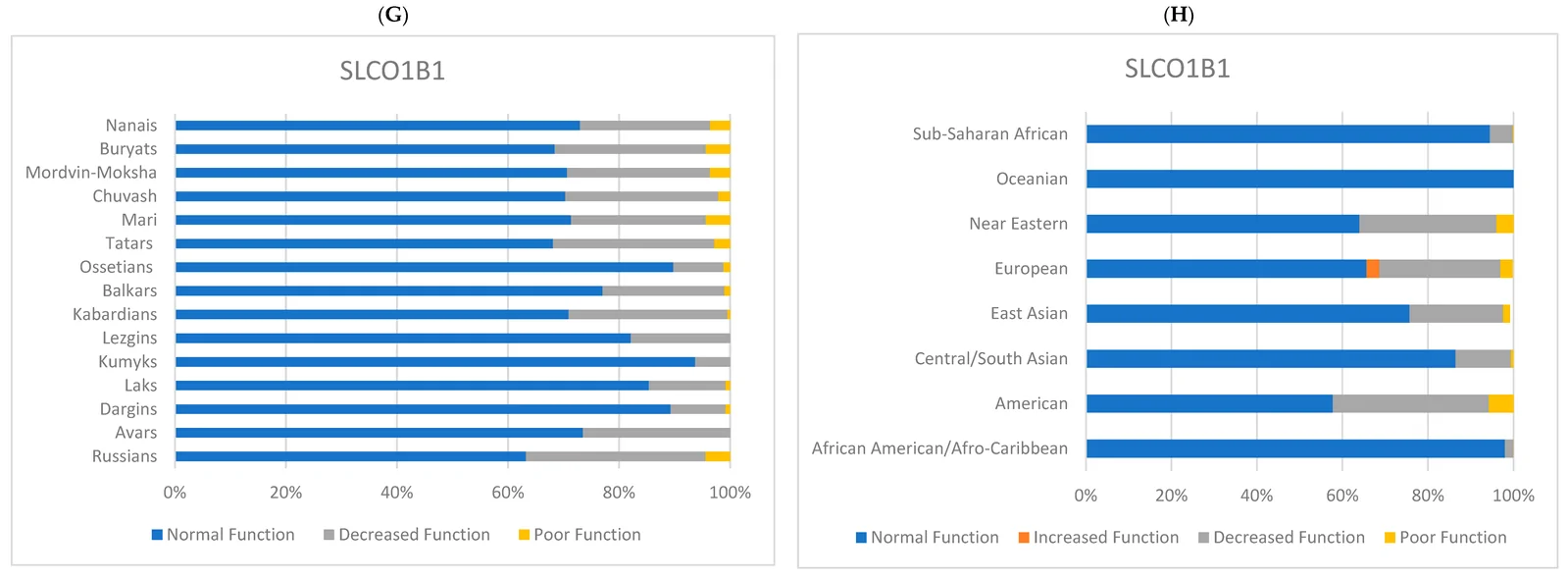
Distribution of CYP2C9, CYP2C19, CYP3A5, and SLCO1B1 metabolic phenotypes in the studied ethnic groups and in the main CPIC/ClinPGx biogeographic groups. (**A**) CYP2C9 phenotypes in the 15 studied ethnic groups; (**B**) CYP2C9 phenotypes in the CPIC/ClinPGx biogeographic groups; (**C**) CYP2C19 phenotypes in the 15 studied ethnic groups; (**D**) CYP2C19 phenotypes in the CPIC/ClinPGx biogeographic groups; (**E**) CYP3A5 phenotypes in the 15 studied ethnic groups; (**F**) CYP3A5 phenotypes in the CPIC/ClinPGx biogeographic groups; (**G**) SLCO1B1 transporter function categories in the 15 studied ethnic groups; (**H**) SLCO1B1 transporter function categories in the CPIC/ClinPGx biogeographic groups. NM—normal metabolizer; IM—intermediate metabolizer; PM—poor metabolizer; UM—ultrarapid metabolizer; RM—rapid metabolizer.

For between-group comparisons, predicted phenotypes were additionally grouped into composite categories reflecting the direction of the functional change (CYP2C9 IM + PM; CYP2C19 UM + RM and IM + PM; CYP3A5 PM; SLCO1B1 decreased + poor function). These are analytical constructs defined for 2 × 2 comparison rather than standardized CPIC phenotypes, and individuals assigned to the same composite category do not necessarily share the same therapeutic recommendation, which remains specific to the gene–drug pair. Grouping was necessary because several individual phenotype categories contained zero or single observations in a number of groups (e.g., CYP2C9 PM in Buryats and Nanais, CYP2C19 UM in Balkars and Nanais, SLCO1B1 poor function in Avars, Kumyks and Lezgins), precluding stable pairwise comparison at that level. The frequencies of all individual CPIC phenotype categories are reported for every ethnic group in Figure 1 and Tables S2–S5.

## 3. Results

### 3.1. General Characterization of Allele Frequencies and Phenotypes

Carriage of 10 pharmacogenetic markers across seven genes (CYP2C9, CYP2C19, CYP3A5, SLCO1B1, VKORC1, CYP4F2, ABCB1; see Table 1) was analyzed in all 15 ethnic groups of the study (*n* = 2304). The complete distribution of allele and genotype frequencies for each group is presented in Table S1 (Supplementary Materials).

Genotype distributions were consistent with the Hardy–Weinberg equilibrium (χ^2^, *p* > 0.05) in 130 of 143 testable marker-group combinations (90.9%); seven combinations were monomorphic and could not be tested. The most pronounced deviations were observed for VKORC1 c.-1639G>A in Tatars (χ^2^ = 52.92; *p* = 3.5 × 10^−13^) and for CYP4F2*3 in Chuvash (χ^2^ = 51.42; *p* = 7.5 × 10^−13^) and Nanais (χ^2^ = 42.37; *p* = 7.6 × 10^−11^); in all three, the expected genotype counts were large (minimum expected count 6.1–12.8), so these deviations are not attributable to sparse categories. Less pronounced deviations were observed for CYP2C9*3 (Tatars), CYP2C19*2 (Kabardians, Balkars), CYP2C19*17 (Balkars), CYP3A5*3 (Avars, Dargins, Lezgins, Nanais), SLCO1B1*5 (Ossetians), and VKORC1 (Chuvash). For CYP3A5*3 the expected count of the minor homozygote was below one in all four groups; on the exact test, the deviation in Nanais was no longer significant (*p* = 0.067 versus χ^2^ *p* = 0.021), while all other combinations gave the same conclusion under both tests (131 of 143 consistent with equilibrium by the exact test). The three most pronounced deviations were all in the direction of heterozygote deficit (observed/expected heterozygotes 0.39–0.53) and, in each affected group, involved one or two markers rather than the panel as a whole, which is more consistent with locus-specific effects than with population substructure or nonrandom mating, neither of which was directly assessed in this study (Table S1).

Interethnic frequency variability differed substantially among markers. The widest range in variant allele frequency was observed for VKORC1 c.-1639G>A (rs9923231): from 29.6% in Tatars to 93.0% in Nanais, reflecting the well-known gradient in VKORC1 A-allele frequency between European and East Asian populations. A similar, though less pronounced, pattern was seen for CYP2C19*3 (rs4986893): the allele was virtually absent in most North Caucasus and Volga-region groups (0–2.7%) but reached 16.2% in Buryats and 9.6% in Nanais–the two groups whose allele frequency profile is closest to that reported for East Asian populations. The opposite pattern was observed for CYP2C19*17 (rs12248560, the increased-function allele): the *17 frequency was lowest in Nanais (1.7%) and Buryats (9.2%) but reached 30.6% in Mari.

Substantial interethnic variability was also characteristic of ABCB1 c.3435C>T (44.7–80.5%), CYP2C9*2 (0–35.4%), and CYP4F2*3 (16.1–50.5%). CYP2C9*3, CYP2C19*2, and SLCO1B1 c.521C showed moderate variability (15.7–18.1%).

The smallest interethnic variability was observed for CYP3A5*3 (rs776746): the frequency of the no-function CYP3A5*3 allele remained high across all 15 groups (87.1–95.7%), without pronounced regional deviations, consistent with the general pattern of the low prevalence of the CYP3A5-expressing phenotype outside populations of African ancestry.

### 3.2. Group-Level Phenotype Distribution and Comparison with Global Data

Comparison of phenotype category frequencies in the studied ethnic groups with the nine CPIC/ClinPGx biogeographic reference groups revealed a consistent pattern: the large majority of groups closely matched the European group across all four genes, whereas the most pronounced and systematic deviations from it were observed in Buryats and Nanais–the only groups in the study with a substantial East Asian ancestry component. The detailed distribution for each gene is presented below (Figure 1A–H).

#### 3.2.1. CYP2C9 (Figure 1A,B)

In the CPIC/ClinPGx European reference group, the proportion of normal metabolizers (NM) is 62.8%, intermediate metabolizers (IM) 34.5%, and poor metabolizers (PM) 2.6%. Most of the studied groups broadly matched this pattern (Russians: 65.4/32.4/2.2%; Ossetians: 64.8/31.1/4.1%; Tatars: 68.1/27.0/4.3%). Lezgins stood out sharply, with the NM proportion reduced to 35.0% and the PM proportion reaching 17.9%–nearly seven times higher than the European reference PM frequency and higher than the highest value among all nine CPIC/ClinPGx biogeographic groups (Central/South Asian, 3.8%). The opposite pattern was observed in Buryats and Nanais: PM was essentially absent (0%) in both groups, and the NM proportion (93.0% and 86.1%, respectively) exceeded even the CPIC/ClinPGx East Asian reference group (NM 83.8%, PM 0.6%). The latter is, in principle, consistent with the reduced frequency of CYP2C9 reduced-function alleles in populations of East Asian ancestry.

#### 3.2.2. CYP2C19 (Figure 1C,D)

The CPIC/ClinPGx European group is characterized by the following distribution: UM 4.6%, RM 27.1%, NM 39.6%, IM 26.2%, PM 2.4%; the East Asian group shows a fundamentally different pattern (UM 0.04%, RM 2.5%, NM 38.1%, IM 46.0%, PM 13.0%). Most of the studied North Caucasus and Volga-region groups matched the European pattern (RM + UM proportion 19–36%). Buryats and Nanais, by contrast, showed a pronounced shift toward the East Asian pattern: the RM + UM proportion was reduced to 10.5% and 1.7%, respectively, with a concurrent increase in IM to 39.5–46.1% and PM to 2.6–7.8%–a pattern that essentially mirrors the East Asian reference group.

#### 3.2.3. CYP3A5 (Figure 1E,F)

Unlike CYP2C9 and CYP2C19, the distribution of CYP3A5 phenotypes showed considerably less intergroup variability, as already noted in Section 3.1. The proportion of poor metabolizers (CYP3A5 non-expressors) ranged from 76.2% (Mari) to 93.2% (Avars), remaining in all 15 groups–including Buryats (77.2%) and Nanais (88.7%)–closer to the CPIC/ClinPGx European frequency (85.7%) than to the East Asian (55.7%) or Sub-Saharan African (27.1%) frequencies. Thus, unlike CYP2C9 and CYP2C19, the East Asian-like pattern observed in Buryats and Nanais for CYP2C9 and CYP2C19 is not accompanied by a marked shift in CYP3A5 phenotype distribution.

#### 3.2.4. SLCO1B1 (Figure 1G,H)

In the CPIC/ClinPGx European group, the distribution is as follows: normal function (NF) 65.6%, decreased function (DF) 28.3%, poor function (PF) 2.9%. Most of the studied groups showed a higher NF proportion than the reference (63.2–93.7%), and the lowest combined proportion of decreased and poor function (DF + PF) was observed in Kumyks (6.3%)–almost 5 times lower than in the European reference group (31.2%). Russians, by contrast, were the only group whose DF + PF proportion (36.8%) exceeded the European reference frequency.

### 3.3. Distribution of Phenotype Categories for CYP2C9, CYP2C19, CYP3A5, and SLCO1B1

**Figure 2:**
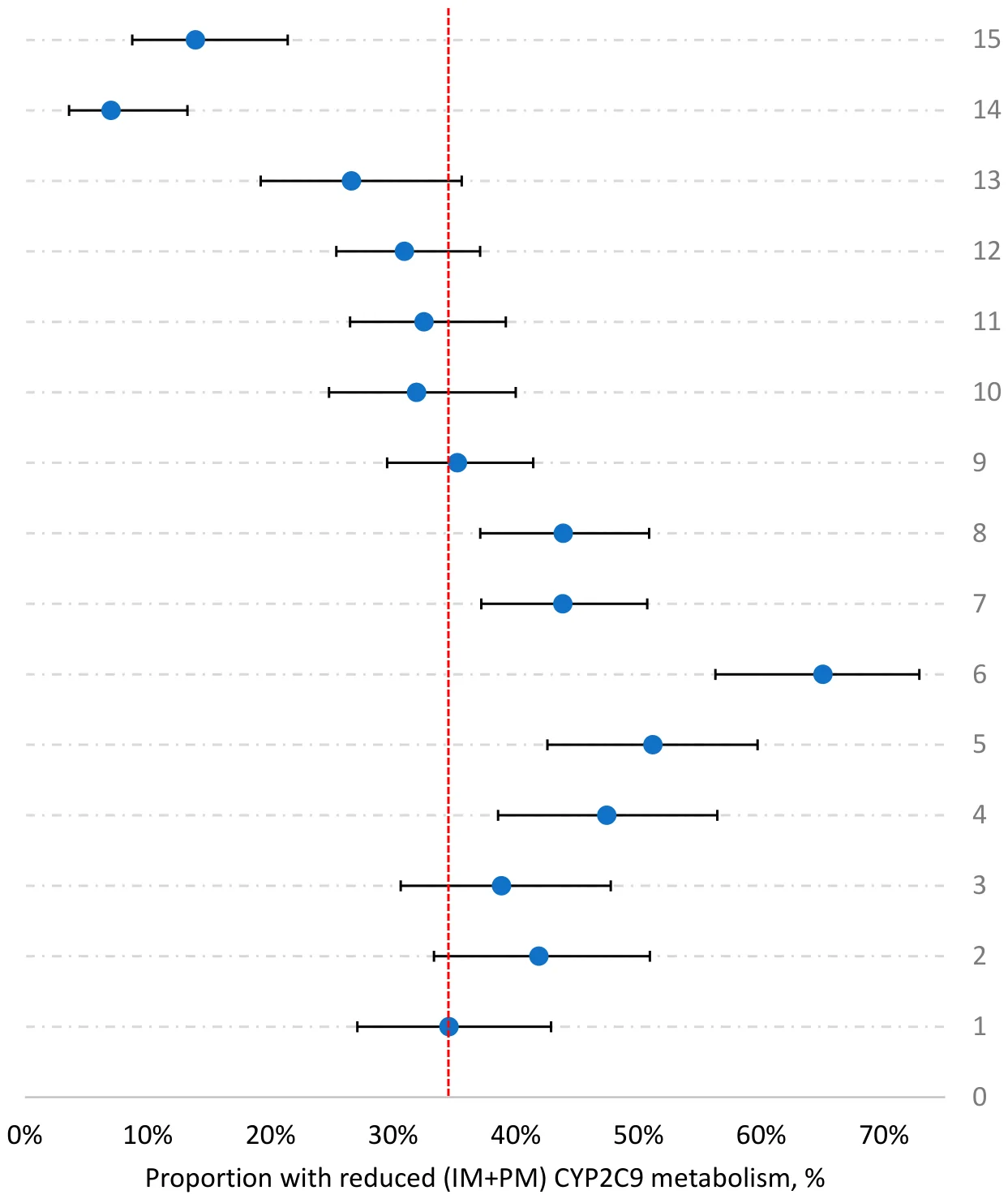
Interethnic distribution of the proportion of carriers of the CYP2C9 reduced-enzyme-activity phenotype (IM + PM). Note: 1–Russians; 2–Avars; 3–Dargins; 4–Laks; 5–Kumyks; 6–Lezgins; 7–Kabardins; 8–Balkars; 9–Ossetians; 10–Tatars; 11–Mari; 12–Chuvash; 13–Moksha Mordvins; 14–Buryats; 15–Nanais.

**Figure 3:**
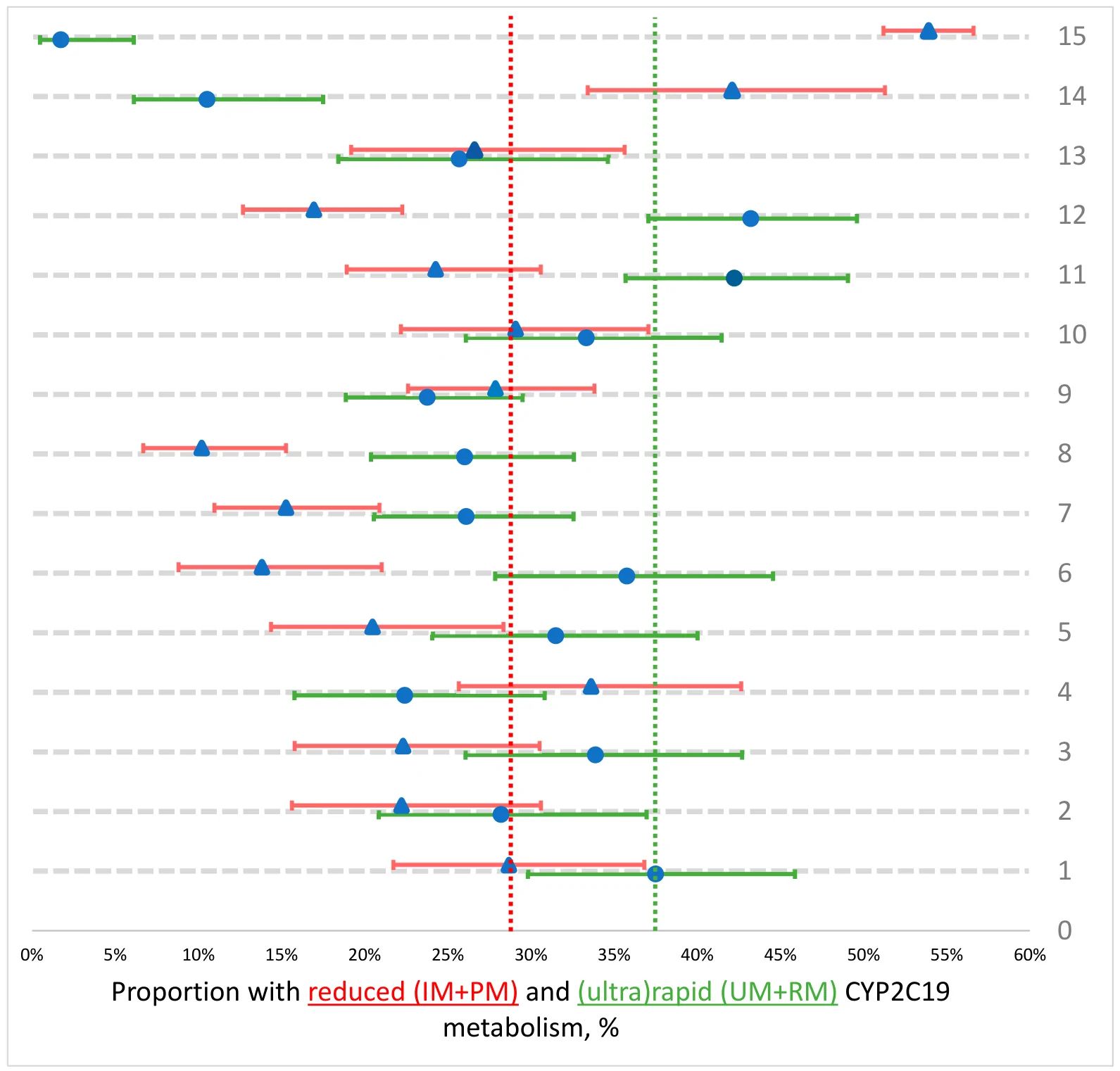
Interethnic distribution of the proportion of carriers of CYP2C19 phenotypes with decreased (IM + PM) and increased (UM + RM) enzyme activity. Note: 1–Russians; 2–Avars; 3–Dargins; 4–Laks; 5–Kumyks; 6–Lezgins; 7–Kabardins; 8–Balkars; 9–Ossetians; 10–Tatars; 11–Mari; 12–Chuvash; 13–Moksha Mordvins; 14–Buryats; 15–Nanais.

**Figure 4:**
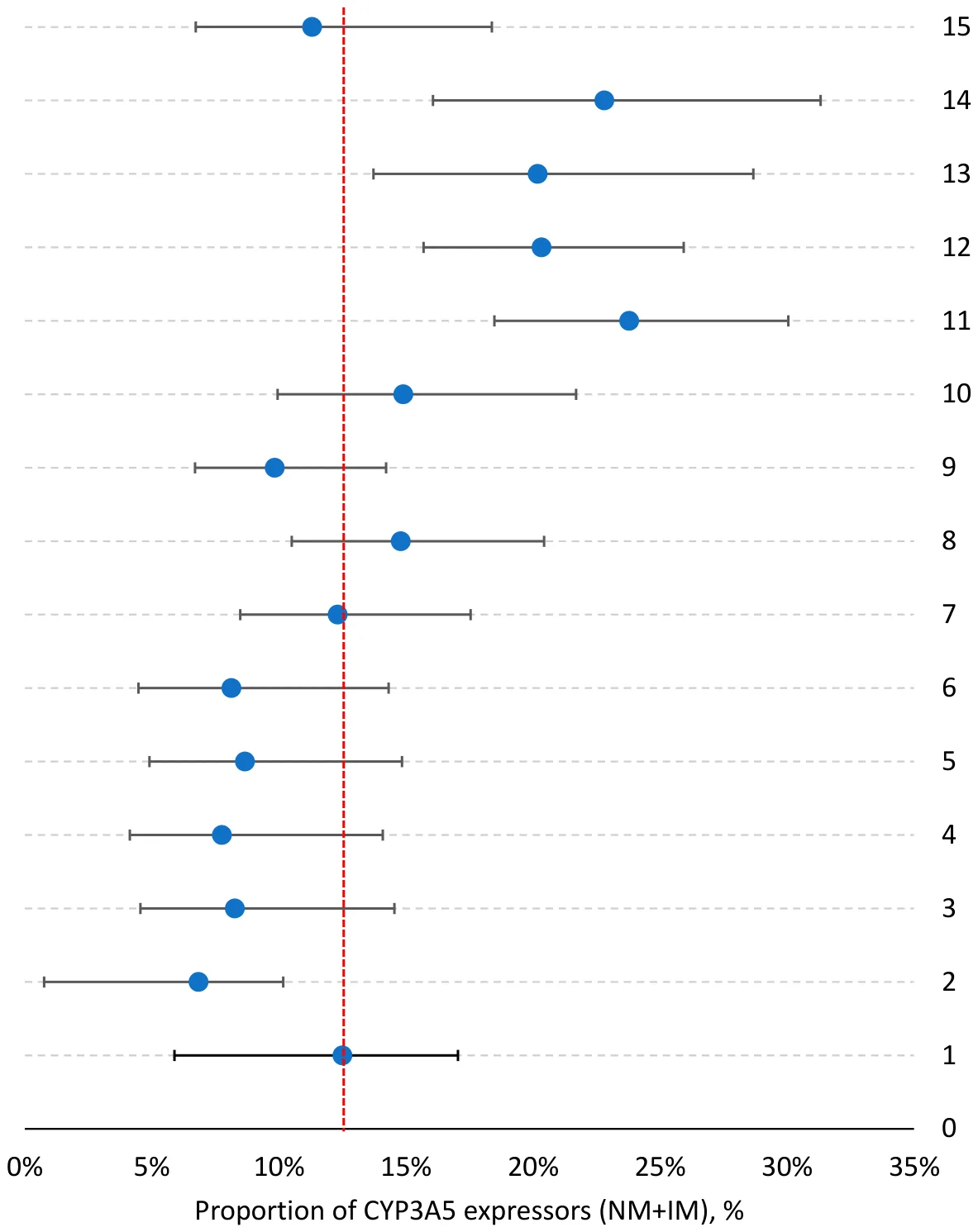
Interethnic distribution of the proportion of carriers of the CYP3A5*3 (rs776746) expressors (NM + IM). Note: 1–Russians; 2–Avars; 3–Dargins; 4–Laks; 5–Kumyks; 6–Lezgins; 7–Kabardins; 8–Balkars; 9–Ossetians; 10–Tatars; 11–Mari; 12–Chuvash; 13–Moksha Mordvins; 14–Buryats; 15–Nanais.

**Figure 5:**
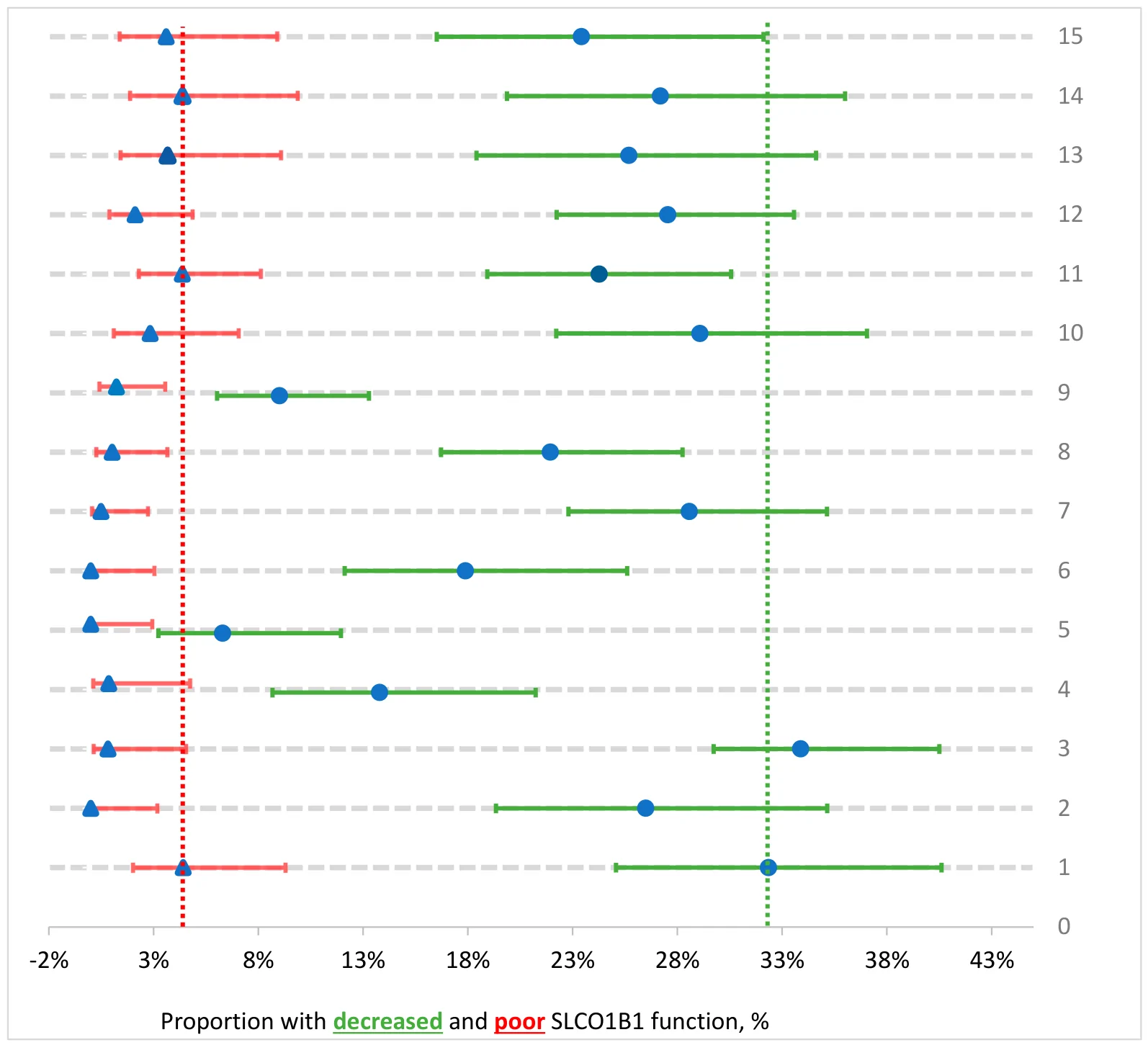
Interethnic distribution of the proportion of carriers of SLCO1B1 phenotypes with decreased and poor transport function. Note: 1–Russians; 2–Avars; 3–Dargins; 4–Laks; 5–Kumyks; 6–Lezgins; 7–Kabardins; 8–Balkars; 9–Ossetians; 10–Tatars; 11–Mari; 12–Chuvash; 13–Moksha Mordvins; 14–Buryats; 15–Nanais.

In the study sample overall (*n* = 2304), the proportion of individuals with intermediate and poor CYP2C9 metabolism (IM + PM) was 36.6% (842/2304); with increased CYP2C19 activity (UM + RM), 29.3% (675/2304); with decreased CYP2C19 activity (IM + PM), 24.4% (563/2304); with the CYP3A5 poor metabolizer phenotype, 86.0% (1982/2304); and with decreased or poor SLCO1B1 function (DF + PF), 23.6% (542/2300). The group-level distribution of these clinically significant categories is presented in Figure 2, Figure 3, Figure 4 and Figure 5.

#### 3.3.1. CYP2C9: Proportion of Intermediate and Poor Metabolizers (IM + PM, Figure 2)

In Russians, the combined IM + PM proportion was 34.6% (47/136). A statistically significantly higher proportion was found in Kumyks–51.2% (65/127; *p*-adj = 0.030)–and, most markedly, in Lezgins–65.0% (80/123; *p*-adj = 7.6 × 10^−6^)–among whom nearly two-thirds of those examined carry the reduced-enzyme-activity phenotype. The opposite pattern was observed in Buryats–7.0% (8/114; *p*-adj = 1.2 × 10^−6^)–and Nanais–13.9% (16/115; *p*-adj = 0.0011)–in whom the IM + PM proportion was 2.5- to 5-fold lower than in Russians.

#### 3.3.2. CYP2C19: Proportion of Decreased-Activity (IM + PM), Ultrarapid, and Rapid Metabolizers (UM + RM, Figure 3)

In Russians, the IM + PM proportion was 28.7% (39/136). A statistically significantly lower proportion was found in Lezgins–13.8% (17/123)–Kabardians–15.3% (31/203)–Balkars–10.2% (20/196)–and Chuvash–16.9% (40/236) (*p*-adj from 0.014 to 2.7 × 10^−4^). In Nanais, by contrast, the IM + PM proportion was statistically significantly increased almost 2-fold–53.9% (62/115; *p*-adj = 4.2 × 10^−4^).

For the proportion of ultrarapid and rapid metabolizers (UM + RM), the figure in Russians was 37.5% (51/136). A statistically significantly lower proportion was observed in Laks–22.4% (26/116; *p*-adj = 0.046), Ossetians–23.8% (58/244; *p*-adj = 0.030), Buryats–10.5% (12/114; *p*-adj = 6.1 × 10^−6^) and Nanais–only 1.7% (2/115; *p*-adj = 1.2 × 10^−12^)–meaning that increased CYP2C19 activity is more than 20 times less frequent in Nanais than in Russians. In no group was the UM + RM proportion statistically significantly higher than the reference.

#### 3.3.3. CYP3A5: Proportion of Expressors (NM + IM, Figure 4)

In Russians, the PM proportion was 87.5% (119/136), corresponding to 12.5% expressors (NM + IM; Figure 4). After correction for multiple comparisons, no statistically significant difference from Russians was found for any of the 14 comparison groups (sample range 76.2–93.2%).

#### 3.3.4. SLCO1B1: Proportion of Decreased and Poor Function (Decreased + Poor Function, Figure 5)

In Russians, the combined DF + PF proportion was 36.8% (50/136)–the highest value among all 15 groups in the study. A statistically significantly lower proportion was found in Kumyks–6.3% (8/127; *p*-adj = 8.7 × 10^−9^)–Ossetians–10.2% (25/244; *p*-adj = 8.7 × 10^−9^)–Dargins–10.7% (13/121; *p*-adj = 5.1 × 10^−6^)–Laks–14.7% (17/116; *p*-adj = 3.3 × 10^−4^)–Lezgins–17.9% (22/123; *p*-adj = 0.0023)–and Balkars–23.0% (45/196; *p*-adj = 0.0158). In no group was the DF + PF proportion statistically significantly higher than in Russians.

The most consistent pattern across several genes simultaneously was observed in Buryats and Nanais: a reduced CYP2C9 IM + PM proportion combined with a reduced CYP2C19 UM + RM proportion (in Nanais, together with a concurrently increased CYP2C19 IM + PM proportion)–a combination not seen in any of the other 15 groups in the study. The complete distribution of all phenotype categories for each gene (including individual phenotype category frequencies) is presented in Tables S2–S5; the results of the statistical comparison of each of the 14 groups with the Russian reference group are presented in Table S6 (Supplementary Materials).

### 3.4. Genotype-Based Expected Warfarin Dose Ranges Derived from CYP2C9 Diplotype, VKORC1 and CYP4F2 Genotypes

The distribution of CYP2C9 diplotype and VKORC1 genotype combinations across the three FDA expected maintenance dose ranges, together with CYP4F2*3 carriage as a modifying factor, is presented in Table 2.

In the study sample overall, the 5–7 mg/day range was expected in 48.5% (1117/2304), the 3–4 mg/day range in 42.8% (985/2304), and the 0.5–2 mg/day range in 8.8% (202/2304). Carriage of the CYP4F2*3 allele, which, within each range, is associated with a 5–10% dose increase, was noted in 47.0% (1084/2304) of those examined.

The most pronounced deviation from the overall sample distribution was observed in Nanais and Buryats: the 5–7 mg/day range was expected in only 13.0% (15/115) and 29.8% (34/114), respectively–3.7-fold and 1.6-fold lower than the sample average–with a concurrent increase in the 3–4 mg/day range to 73.9% (85/115) and 69.3% (79/114), the two highest values among all 15 groups. In Buryats the 0.5–2 mg/day range was almost absent (0.9%, 1/114), reflecting the near-absence of CYP2C9 no-function alleles in this group; in Nanais, by contrast, it reached 13.0% (15/115), as the high VKORC1 A-allele frequency places CYP2C9*1/*3 carriers in this group into the lowest dose range. The opposite pattern was seen in Mordvin-Moksha (67.0%, 73/109), Tatars (63.8%, 90/141) and Russians (62.5%, 85/136), in whom the proportion expected to require 5–7 mg/day exceeded the sample average.

The proportion expected to require 0.5–2 mg/day was highest in Lezgins, 21.1% (26/123), approximately 2.4 times the sample average (8.8%) and the highest of all 15 groups, with the next highest being Avars at 15.4% (18/117) and the lowest Buryats at 0.9% (1/114). This follows from the maximal CYP2C9 IM + PM proportion in Lezgins (65.0%, Section 3.3).

Carriage of the CYP4F2*3 allele was most common in Balkars–77.0% (151/196)–making this group distinctive not only in allele frequency but also in the overall proportion of individuals for whom a 5–10% upward dose adjustment within the assigned range would be indicated. The lowest proportion requiring the modifier was seen in Chuvash–23.3% (55/236) and Mari–32.0% (66/206).

## 4. Discussion

This study provides the first characterization of the distribution of the pharmacogenetically significant markers CYP2C9, CYP2C19, CYP3A5, SLCO1B1, VKORC1, CYP4F2, and ABCB1 across 15 ethnic groups of Russia, totaling 2304 individuals. The results demonstrated that the degree of interethnic variability differed substantially among genes, being the greatest for VKORC1 and the smallest for CYP3A5. Formal statistical comparison revealed two categories of deviation from the Russian reference group that differ fundamentally in origin: first, a systematic shift concordant across several genes in Buryats and Nanais (reduced CYP2C9 IM + PM and CYP2C19 UM + RM proportions together with an increased CYP2C19 IM + PM proportion), consistent with the direction expected for populations with an East Asian ancestry component; and second, isolated but statistically pronounced deviations in individual North Caucasus groups–most notably in Lezgins (the highest CYP2C9 IM + PM proportion in the entire sample) and in Kumyks, Ossetians, and Dargins (the lowest proportion of reduced SLCO1B1 function)–which do not form a common geographic or linguistic pattern. These differences are reflected in an uneven distribution of genotype-based expected warfarin dose ranges among groups, including a shift toward the 3–4 mg/day range in Buryats and Nanais alongside the highest proportion expected to require 0.5–2 mg/day in Lezgins.

On one hand, these findings show that using uniform, nationally averaged frequencies to estimate expected pharmacogenetic risk–in the absence of individual genotyping–may yield substantially biased estimates for individual ethnic groups, systematically underestimating risk in some groups and overestimating it in others. On the other hand, such data are a necessary input to identifying the regions where implementation of genotyping should begin, which is particularly relevant in regions where specific ethnic groups live in compact settlements.

The most pronounced and clinically significant deviation from the overall sample was observed in Buryats and Nanais, in whom a reduced CYP2C9 IM + PM proportion was combined with a reduced CYP2C19 UM + RM proportion and a concurrently increased CYP2C19 IM + PM proportion. Concordance in the direction of deviation across two independently located genomic loci is consistent with what has been reported for populations sharing a common ancestry component. Published population-genetic studies describe an East Asian ancestry component in Buryat and Nanai populations [25]. The phenotype distribution obtained for these two groups is broadly consistent with previously published data on other Siberian and East Asian-descended peoples of Russia, including studies of Nanais [26] and Yakuts [27], which likewise reported a shift in pharmacogenetic profile toward the pattern characteristic of East Asian populations.

The deviations recorded in individual North Caucasus groups are of a different nature–most notably in Lezgins, who showed the highest CYP2C9 IM + PM proportion in the entire sample (65.0%). Unlike in Buryats and Nanais, here, the deviation affects a single gene and is not reproduced in either neighboring or linguistically related peoples of the same region (Avars, Dargins, Kumyks). One possible interpretation of such an isolated, non-systemic pattern relates to the demographic history of the mountainous districts of Dagestan, where a traditionally high rate of within-ethnicity and within-village marriage, combined with the relatively small size of individual communities, has been proposed to increase the role of genetic drift and founder effects in shaping the allele frequency spectrum of a group; this feature has been described in independent studies of the population of Dagestan [28]. The relatively high CYP2C9 IM + PM frequency in Lezgins shows that the deviation is specific to this group and to this gene and is not reproduced in neighboring or linguistically related peoples of the same region.

CYP2C9 encodes an enzyme responsible for the metabolism of several drug classes: nonsteroidal anti-inflammatory drugs (NSAIDs), warfarin, and phenytoin, for which the IM and PM phenotypes are associated with reduced substrate clearance, although the resulting recommendation is specific to the drug rather than common to the class: a genotype-based dose range for warfarin, a reduced starting dose for phenytoin, and drug-specific guidance for individual NSAIDs [29,30], as well as several statins (fluvastatin in particular), for which CYP2C9 genotype, together with SLCO1B1 and ABCG2, is included in the joint guideline on the risk of statin-associated musculoskeletal symptoms [31]. In Russians, the proportion of reduced-metabolism phenotypes (IM + PM) was 34.6%; a statistically significantly higher proportion was found in Kumyks and Lezgins (*p*-adj = 0.030 and 7.6 × 10^−6^, respectively), whereas in Buryats and Nanais, the proportion was statistically significantly lower (*p*-adj = 1.2 × 10^−6^ and 0.0011, respectively). Thus, the proportion of individuals for whom a CYP2C9 genotype-guided dosing recommendation would apply for at least one of the substrates listed above is statistically significantly higher in Lezgins than in Russians, and significantly lower in Buryats and Nanais. As with the overall CYP2C9 IM + PM proportion noted above, the relatively high proportion of the reduced-function phenotype in Lezgins is localized rather than regional: it is not accompanied by a comparable deviation in any other North Caucasus group and could not, accordingly, have been predicted in advance.

CYP2C19 is involved in the metabolism and bioactivation of the antiplatelet prodrug clopidogrel into its active metabolite, in the metabolism of proton pump inhibitors (PPIs), and in the metabolism of several selective serotonin reuptake inhibitor antidepressants and related agents (citalopram, escitalopram, sertraline, and others). The clinical consequence of a given CYP2C19 phenotype is drug-dependent and cannot be assigned a single direction of risk. For clopidogrel, reduced activity (IM, PM) decreases formation of the active metabolite and is associated with an increased risk of recurrent cardiovascular events on standard antiplatelet therapy [32]. For PPIs, reduced activity increases systemic exposure and generally enhances acid suppression, whereas increased activity (RM, UM) lowers exposure and is the phenotype associated with a risk of inadequate therapeutic effect [33]. For CYP2C19-metabolized antidepressants, the direction depends on the specific agent: reduced activity increases exposure and the probability of concentration-dependent adverse effects, for which CPIC recommends dose reduction or an alternative agent, whereas increased activity may result in subtherapeutic concentrations and non-response [34].

Against this background, the proportion of increased-activity phenotypes (UM, RM) in Russians was 37.5%; a statistically significantly lower proportion was found in Laks, Ossetians, Buryats, and, most markedly, Nanais (*p*-adj = 0.046, 0.030, 6.1 × 10^−6^, and 1.2 × 10^−12^, respectively). In Nanais, increased activity is more than 20 times less frequent than in Russians; the number of individuals expected to be at risk of inadequate PPI effect or of antidepressant non-response through rapid metabolism is therefore lower, not higher, in these groups than in Russians. The proportion of reduced-activity CYP2C19 phenotypes (IM + PM) in Russians was 28.7%; it was statistically significantly lower in Lezgins, Kabardians, Balkars, and Chuvash but statistically significantly higher in Nanais (*p*-adj = 4.2 × 10^−4^), in whom the proportion of individuals with an expected risk of reduced clopidogrel efficacy is almost twice that in Russians and in whom a correspondingly larger proportion would be expected to show increased exposure to PPIs and to CYP2C19-metabolized antidepressants. The direction of this deviation in Buryats and Nanais matches that already noted for CYP2C9, indicating that the shift involves more than a single locus rather than being an isolated finding specific to one gene.

CYP3A5 determines the metabolism of tacrolimus, an immunosuppressant used after organ transplantation; carriers of the normal or intermediate metabolizer phenotype (CYP3A5 expressors) require a higher starting dose to achieve therapeutic concentrations compared with poor metabolizers [35]. In Russians, the PM proportion was 87.5%, and no statistically significant difference from this value was found for any of the 14 comparison groups after correction. Of all the genes examined, CYP3A5 showed the most homogeneous distribution relative to the reference group, providing no grounds for an “ethnically differentiated” approach to initial tacrolimus dose selection in the studied groups.

SLCO1B1 encodes the OATP1B1 transporter, which mediates hepatic uptake of statins; reduced or absent transporter function increases systemic statin exposure and is associated with an increased risk of statin-associated musculoskeletal symptoms, including, rarely, rhabdomyolysis [31]. In Russians, the combined proportion with decreased and poor transporter function was 36.8%–the highest value among all 15 groups. In Dargins, Kumyks, Ossetians, Laks, Lezgins, and Balkars, this proportion was statistically significantly lower (*p*-adj < 0.05), and in no group was it statistically significantly higher than in Russians. Thus, the Russian group showed the highest genetic risk frequency of the assayed reduced-function SLCO1B1 marker among the 15 groups studied. Unlike the patterns established for CYP2C9 and CYP2C19, this deviation does not align with either the East Asian or the Dagestan pattern and is locus-specific, whatever its underlying cause: the direction and magnitude of interethnic differences in the studied samples cannot be reliably extrapolated from one gene to another, even within the same group.

ABCB1 encodes P-glycoprotein, an efflux transporter with a broad substrate spectrum that includes the direct oral anticoagulants (dabigatran, rivaroxaban, apixaban), clopidogrel, several antidepressants, and PPIs [36]. The marker rs1045642 (c.3435C>T) is a synonymous substitution; however, as shown by Kimchi-Sarfaty et al. (2007), it appears to alter translation rate and, consequently, protein conformation and substrate specificity, without changing mRNA or protein levels [37]. The accumulated evidence across individual substrates is heterogeneous: for antiplatelet therapy, ABCB1 genotype was associated with cardiovascular outcomes independently of CYP2C19 in the TRITON-TIMI 38 study [38], whereas for dabigatran, the results of systematic reviews are inconsistent, and the variant more consistently associated with drug exposure was not rs1045642 itself but the linked intronic variant rs4148738 [39,40]. Given this heterogeneity of effect across P-glycoprotein substrates, the ABCB1 rs1045642 marker frequency obtained in the present study is, at this stage, primarily of interest as a basis for subsequent research on each of the drug classes listed above individually.

Interethnic differences in expected warfarin dose driven by CYP2C9, VKORC1, and CYP4F2 frequencies have by now been described for many regions of the world: individuals of Asian ancestry on average require a lower dose than those of European ancestry, while individuals of African ancestry require higher doses [41]. Such differences persist even within a single geographic region, for example, among Malays, Chinese, and Indians in Southeast Asia [42]. At the same time, the contribution of VKORC1 c.-1639G>A to warfarin dose variability differs substantially among populations: according to the International Warfarin Pharmacogenetics Consortium (IWPC), this polymorphism explains the largest share of dose variability in individuals of European ancestry and a markedly smaller share in individuals of Asian and African ancestry, while the final dose in each of the three groups is equally determined by the joint contribution of VKORC1 and CYP2C9 [41]. The high frequency of the VKORC1 A allele (81.6–93.0%) combined with a low frequency of CYP2C9 reduced-function alleles shifts the expected dose distribution toward the 3–4 mg/day range in these groups (73.9% in Nanais, 69.3% in Buryats). This may indicate that standard dosing algorithms developed in European cohorts could systematically overestimate the required dose in these groups. In Lezgins, by contrast, the proportion expected to require 0.5–2 mg/day (21.1%) was the highest of all 15 groups and approximately 2.4 times the sample average (8.8%), which, combined with the high CYP2C9 IM + PM proportion (65.0%), supports the value of testing before prescribing warfarin. The overwhelming majority of published studies on warfarin pharmacogenetics have been conducted in cohorts of European or East Asian ancestry, whereas populations of the Caucasus, the Volga region, and Siberia remain extremely underrepresented in this literature [43]. In this context, the differences established here extend the existing evidence base to previously undescribed population groups of Russia in the warfarin pharmacogenetics literature.

The frequency of a single clinically significant phenotype in a population is not, by itself, sufficient grounds for formulating national or regional testing-implementation recommendations. Economic evaluations of pharmacogenetic testing generally favor its use: more than half of the evaluations covering gene–drug pairs on the FDA biomarker list, and of those covering pairs with CPIC guidelines, support testing, and a substantial minority find it cost-saving [2,3]. The expected return depends in part on how frequently actionable phenotypes occur in the population tested, since fewer individuals need to be genotyped to identify one person for whom a guideline-based change in therapy applies when that frequency is higher. The differences documented here therefore bear on the expected efficiency of testing between groups. In Lezgins, 65.0% of individuals carry a CYP2C9 intermediate or poor metabolizer phenotype against 34.6% in Russians; in Buryats and Nanais, the expected warfarin dose distribution is shifted away from the range assumed by algorithms developed in European cohorts. Conversely, for genes with a low actionable frequency in a given group, panel-wide testing would be correspondingly less efficient for that gene. Frequency is only one input: economic viability also depends on panel composition, drug acquisition costs and local prescribing patterns, as shown for preemptive panel testing in the multiethnic population of Singapore [44], and none of these was assessed here. The present study provides the underlying population-level data on ethnic groups of Russia needed for such an assessment, but it does not substitute for the subsequent economic and organizational analysis required to translate the established differences into specific regional recommendations.

Within these constraints, the results of this study have two complementary practical implications: optimization of therapy at the individual patient level and rationalization of the implementation of testing at the healthcare-system level. A comparable distinction was proposed when constructing a similarly purposed panel of pharmacogenetic biomarkers in several European countries [9]. At the individual level, an unconditional rule remains: ethnic affiliation, even when documented, does not substitute for the result of a genetic test for any of the genes examined, and its use as a clinical surrogate is already limited by the heterogeneity of ethnic self-identification criteria [45]. At the healthcare-system level, the differences established here identify the groups in which the population-level burden of actionable phenotypes departs most from the national average: the markedly elevated proportion of clinically significant CYP2C9 phenotypes in Lezgins and the systematic shift in warfarin dose categories in Buryats and Nanais. Whether these groups would also yield the greatest return from preemptive testing cannot be determined from frequency data alone and would require the additional inputs noted above. The clinical utility of preemptive panel genotyping as such is supported by the prospective, multicenter PREPARE study, in which preemptive genotyping of a gene panel including CYP2C19 and CYP3A5 reduced the incidence of clinically relevant adverse drug reactions by 30% compared with standard care [46]. A similar principle–matching pharmacogenetic marker frequencies to local clinical and economic conditions to select priority gene–drug pairs–is already being applied when planning the implementation of testing in countries with pronounced ethnic population heterogeneity [47,48].

This study has several limitations. First, participants were assigned to an ethnic group based on self-identification rather than genetic ancestry data, a widely accepted but not error-free practice in population pharmacogenetics [8]. Participants were enrolled as unrelated individuals on the basis of self-reported absence of family relationship to other participants; relatedness was not verified genetically. Second, ethnic groups were recruited in specific regions where the corresponding ethnicity or people traditionally resides in compact settlements. Third, the marker panel included the clinically most significant and best-studied allelic variants (CYP2C9*2/*3, CYP2C19*2/*3/*17, and others) but did not cover rare or population-specific alleles that may exist specifically in small, isolated groups. Fourth, haplotype phase was likewise not determined, so diplotypes for individuals heterozygous at two positions rest on the in-trans assumption; this affected 60 individuals for CYP2C9 and eight for CYP2C19 and the assigned warfarin dose range in 33 individuals. Finally, this study is limited to allele, genotype, predicted phenotype, and dose-category frequencies; it does not include data on actual clinical outcomes in the studied groups. The distribution obtained should be regarded as a basis for prioritizing further research–including clinical studies–and the implementation of genotyping, rather than as direct evidence of differences in clinical outcomes between groups.

## 5. Conclusions

This study characterized the distribution of ten pharmacogenetic markers across seven genes (CYP2C9, CYP2C19, CYP3A5, SLCO1B1, VKORC1, CYP4F2, and ABCB1) across 15 ethnic groups of Russia, totaling 2304 individuals. The results demonstrate statistically significant interethnic differences in allele and genotype frequencies, with the degree of variability differing substantially among genes from minimal for CYP3A5 to maximal for VKORC1. These findings become more pronounced at the level of clinically significant composite phenotype categories: statistically significant differences from the Russian reference group were established for CYP2C9 in four of 14 groups, for CYP2C19 in eight of 14 groups across both directions of activity, and for SLCO1B1 in six of 14 groups, affecting the efficacy and safety of several widely used drug classes: nonsteroidal anti-inflammatory drugs, phenytoin, statins, clopidogrel, proton pump inhibitors, and antidepressants. Pronounced interethnic differences were also found in the distribution of genotype-based expected warfarin maintenance dose ranges, assigned from CYP2C9 diplotype combined with VKORC1 genotype. These data provide a population-level basis that could form one component of a future framework for prioritizing pharmacogenetic implementation in Russia; however, they do not themselves establish which regions or ethnic groups should receive priority for preemptive testing. This issue further requires data on drug utilization, clinical outcomes, costs and implementation infrastructure.

## Figures and Tables

**Table 1:** Panel of pharmacogenetic markers included in the study and their clinical significance according to AMP and CPIC.

Gene	Marker (*Allele)	rs (dbSNP)	Substitution	CPIC Allele Functional Status	AMP Tier Classification	CPIC Drug–Gene Pairs
CYP2C9	*2	rs1799853	c.430C>T	Decreased function	Tier 1: CYP2C9*2, CYP2C9*3, CYP2C9*5, CYP2C9*6, CYP2C9*8, CYP2C9*11; Tier 2: CYP2C9*12, CYP2C9*13, CYP2C9*15	1. NSAIDs; 2. Phenytoin; 3. Warfarin; 4. Statins
	*3	rs1057910	c.1075A>C	Decreased function		
CYP2C19	*2	rs4244285	c.681G>A	No function	Tier 1: CYP2C19*2, CYP2C19*3, CYP2C19*17; Tier 2: CYP2C19*4, *5, *6, *7, *8, *9, *10, *35	1. Clopidogrel; 2. PPIs; 3. Voriconazole; 4. Tricyclic antidepressants; 5. Selective serotonin reuptake inhibitor antidepressants
	*3	rs4986893	c.636G>A	No function		
	*17	rs12248560	c.-806C>T	Increased function		
CYP3A5	*3	rs776746	c.219-237A>G	No function	Tier 1: CYP3A5*3, CYP3A4*22, CYP3A5*6, CYP3A5*7; Tier 2: CYP3A4*20	Tacrolimus
SLCO1B1	*5	rs4149056	c.521T>C	Decreased function	-	Statins
CYP4F2	*3	rs2108622	c.1297G>A	Decreased function	Tier 2: CYP4F2*3, rs12777823, rs61742245, rs72547529.	Warfarin
VKORC1	-	rs9923231	c.-1639G>A	NA	Tier 1: rs9923231 (VKORC1)	Warfarin
ABCB1	-	rs1045642	c.3435C>T	NA	Not classified (exploratory marker)	Not classified (exploratory marker)

Note: ABCB1 rs1045642 is not assigned to an AMP tier and has no CPIC drug–gene pair; it was included as an exploratory marker and is reported descriptively only. * is standard designation for pharmacogenetic markers.

**Table 2:** Distribution of genotype-based expected maintenance warfarin dose ranges, assigned from CYP2C9 diplotype combined with VKORC1 c.-1639G>A genotype according to the FDA-approved prescribing information.

	Ethnic Group	Sample Size, *n*	High 5–7 mg/day	Intermediate 3–4 mg/day	Low 0.5–2 mg/day	CYP4F2*3 Carriers (Modifying Factor) *
1	Russians	136	85 (62.5%)	45 (33.1%)	6 (4.4%)	63 (46.7%)
2	Avars	117	51 (43.6%)	48 (41.0%)	18 (15.4%)	64 (54.7%)
3	Dargins	121	66 (54.5%)	45 (37.2%)	10 (8.3%)	54 (44.6%)
4	Laks	116	51 (44.0%)	49 (42.2%)	16 (13.8%)	65 (56.0%)
5	Kumyks	127	51 (40.2%)	63 (49.6%)	13 (10.2%)	61 (48.0%)
6	Lezgins	123	39 (31.7%)	58 (47.2%)	26 (21.1%)	63 (51.2%)
7	Kabardians	203	97 (47.8%)	89 (43.8%)	17 (8.4%)	91 (44.8%)
8	Balkars	196	92 (46.9%)	86 (43.9%)	18 (9.2%)	151 (77.0%)
9	Ossetians	244	127 (52.0%)	93 (38.1%)	24 (9.8%)	128 (52.5%)
10	Chuvash	236	138 (58.5%)	87 (36.9%)	11 (4.7%)	55 (23.3%)
11	Tatars	141	90 (63.8%)	40 (28.4%)	11 (7.8%)	50 (35.5%)
12	Mari	206	108 (52.4%)	86 (41.7%)	12 (5.8%)	66 (32.0%)
13	Mordvin-Moksha	109	73 (67.0%)	32 (29.4%)	4 (3.7%)	57 (52.3%)
14	Buryats	114	34 (29.8%)	79 (69.3%)	1 (0.9%)	68 (59.6%)
15	Nanais	115	15 (13.0%)	85 (73.9%)	15 (13.0%)	48 (41.7%)
	Total	2304	1117 (48.5%)	985 (42.8%)	202 (8.8%)	1084 (47.1%)

Note: * CYP4F2*3 (rs2108622) carriage is reported separately as a modifying factor: per the CPIC guideline, carriage of the variant allele is associated with a 5–10% dose increase within the category already defined by CYP2C9/VKORC1 and does not reassign individuals between categories.

## Data Availability

The datasets generated and analyzed during this study are not publicly available due to ethical restrictions and patient confidentiality protections under Russian Federation laws on personal data protection (Federal Law No. 152-FZ). However, anonymized data supporting the findings may be made available upon reasonable request from qualified researchers. Requests should include a detailed research proposal and data protection plan.

## Supplementary Materials

The following supporting information can be downloaded at: https://www.mdpi.com/article/10.3390/genes17101275/s1. Table S1: Allele and genotype frequencies of the CYP2C9, CYP2C19, CYP4F2, VKORC1, CYP3A5, SLCO1B1, and ABCB1 markers in 15 ethnic groups of Russia, with assessment of the Hardy–Weinberg equilibrium (χ^2^ test). Table S2: Distribution of CYP2C9 phenotypes (Normal, Intermediate, Poor Metabolizer) in 15 ethnic groups of Russia, with 95% CIs. Table S3: Distribution of CYP2C19 phenotypes (Ultrarapid, Rapid, Normal, Intermediate, Poor Metabolizer) in 15 ethnic groups of Russia, with 95% CIs. Table S4: Distribution of CYP3A5 phenotypes (Normal, Intermediate, Poor Metabolizer) in 15 ethnic groups of Russia, with 95% CIs. Table S5: Distribution of SLCO1B1 phenotypes (Normal, Decreased, Poor Function) in 15 ethnic groups of Russia, with 95% CIs. Table S6: Comparison of frequencies of clinically significant composite phenotype categories of CYP2C9, CYP2C19, CYP3A5, and SLCO1B1 between each of the 14 ethnic groups and the Russian reference group (2 × 2 Fisher’s exact test, Benjamini–Hochberg-corrected).

## Abbreviations

The following abbreviations are used in this manuscript:

AMP	Association for Molecular Pathology
CI	Confidence Interval
ClinPGx	Clinical Pharmacogenomics (formerly PharmGKB)
CPIC	Clinical Pharmacogenetics Implementation Consortium
DF	Decreased Function (SLCO1B1 phenotype)
DNA	Deoxyribonucleic Acid
DPWG	Dutch Pharmacogenetics Working Group
EDTA	Ethylenediaminetetraacetic Acid
FDA	U.S. Food and Drug Administration
FDR	False Discovery Rate
IM	Intermediate Metabolizer
IWPC	International Warfarin Pharmacogenetics Consortium
NF	Normal Function (SLCO1B1 phenotype)
NM	Normal Metabolizer
NSAIDs	Nonsteroidal Anti-Inflammatory Drugs
OATP1B1	Organic Anion Transporting Polypeptide 1B1
PCR	Polymerase Chain Reaction
PF	Poor Function (SLCO1B1 phenotype)
PM	Poor Metabolizer
PPIs	Proton Pump Inhibitors
RM	Rapid Metabolizer
SNP	Single Nucleotide Polymorphism
UM	Ultrarapid Metabolizer
