# Supplementary material for: From Frequencies to Priorities: Interethnic Distribution of Pharmacogenetic Phenotypes as a Basis for Prioritizing Preemptive Testing in Russia’s Multiethnic Population
Source: Genes (Basel). 2026 Oct 14;17(10):1275. doi: 10.3390/genes17101275 (PMC13704235; doi:10.3390/genes17101275)
Supplement: Supplementary file 1 [file genes-17-01275-s001.zip › Table S2.pdf]

Table S2: Distribution of CYP2C9 phenotypes (Normal, Intermediate, Poor Metabolizer) in 15 ethnic groups of Russia, with 95% CIs

#	Ethnic groups	Sample size, n	Normal Metabolizer				Intermediate Metabolizer				Poor Metabolizer			
			n	%	CI lower, %	CI upper, %	n	%	CI lower, %	CI upper, %	n	%	CI lower, %	CI upper, %
1	Russians	136	89	65.4%	57.1%	72.9%	44	32.4%	25.1%	40.6%	3	2.2%	0.8%	6.3%
2	Avars	117	68	58.1%	49.1%	66.7%	35	29.9%	22.4%	38.7%	14	12.0%	7.3%	19.1%
3	Dargins	121	74	61.2%	52.3%	69.4%	42	34.7%	26.8%	43.5%	5	4.1%	1.8%	9.3%
4	Laks	116	61	52.6%	43.6%	61.4%	42	36.2%	28.0%	45.3%	13	11.2%	6.7%	18.2%
5	Kumyks	127	62	48.8%	40.3%	57.4%	61	48.0%	39.5%	56.7%	4	3.1%	1.2%	7.8%
6	Lezgins	123	43	35.0%	27.1%	43.7%	58	47.2%	38.6%	55.9%	22	17.9%	12.1%	25.6%
7	Kabardians	203	114	56.2%	49.3%	62.8%	78	38.4%	32.0%	45.3%	11	5.4%	3.1%	9.4%
8	Balkars	196	110	56.1%	49.1%	62.9%	71	36.2%	29.8%	43.2%	15	7.7%	4.7%	12.2%
9	Ossetians	244	158	64.8%	58.6%	70.5%	76	31.1%	25.7%	37.2%	10	4.1%	2.2%	7.4%
10	Tatars	141	96	68.1%	60.0%	75.2%	38	27.0%	20.3%	34.8%	7	5.0%	2.4%	9.9%
11	Mari	206	139	67.5%	60.8%	73.5%	62	30.1%	24.2%	36.7%	5	2.4%	1.0%	5.6%
12	Chuvash	236	163	69.1%	62.9%	74.6%	67	28.4%	23.0%	34.5%	6	2.5%	1.2%	5.4%
13	Mordvin-Moksha	109	80	73.4%	64.4%	80.8%	27	24.8%	17.6%	33.6%	2	1.8%	0.5%	6.4%
14	Buryats	114	106	93.0%	86.8%	96.4%	8	7.0%	3.6%	13.2%	0	0.0%	0.0%	3.3%
15	Nanais	115	99	86.1%	78.6%	91.3%	16	13.9%	8.7%	21.4%	0	0.0%	0.0%	3.2%

Note: NM – Normal Metabolizer; IM – Intermediate Metabolizer; PM – Poor Metabolizer (CYP2C9 phenotype categories assigned per CPIC diplotype–phenotype tables); n – number of individuals in the given phenotype category; % – phenotype frequency; CI lower, % / CI upper, % – lower and upper bounds of the 95% Wilson confidence interval.
