# Supplementary material for: From Frequencies to Priorities: Interethnic Distribution of Pharmacogenetic Phenotypes as a Basis for Prioritizing Preemptive Testing in Russia’s Multiethnic Population
Source: Genes (Basel). 2026 Oct 14;17(10):1275. doi: 10.3390/genes17101275 (PMC13704235; doi:10.3390/genes17101275)
Supplement: Supplementary file 1 [file genes-17-01275-s001.zip › Table S3.pdf]

Table S3: Distribution of CYP2C19 phenotypes (Ultrarapid, Rapid, Normal, Intermediate, Poor Metabolizer) in 15 ethnic groups of Russia, with 95% CIs.

#	Ethnic groups	Sample size, n	Ultrarapid Metabolizer				Rapid Metabolizer				Normal Metabolizer				Intermediate Metabolizer				Poor Metabolizer			
			n	%	CI lower, %	CI upper, %	n	%	CI lower, %	CI upper, %	n	%	CI lower, %	CI upper, %	n	%	CI lower, %	CI upper, %	n	%	CI lower, %	CI upper, %
1	Russians	136	11	8.1%	4.6%	13.9%	40	29.4%	22.4%	37.6%	46	33.8%	26.4%	42.1%	36	26.5%	19.8%	34.5%	3	2.2%	0.8%	6.3%
2	Avars	117	4	3.4%	1.3%	8.5%	29	24.8%	17.8%	33.3%	58	49.6%	40.7%	58.5%	25	21,4%	14,9%	29,6%	1	0,9%	0,2%	4,7%
3	Dargins	121	6	5.0%	2.3%	10.4%	35	28.9%	21.6%	37.6%	53	43.8%	35.3%	52.7%	26	21,5%	15,1%	29,6%	1	0,8%	0,1%	4,5%
4	Laks	116	5	4.3%	1.9%	9.7%	21	18,1%	12,2%	26,1%	51	44.0%	35.3%	53.0%	37	31,9%	24,1%	40,8%	2	1.7%	0.5%	6.1%
5	Kumyks	127	2	1.6%	0.4%	5.6%	38	29.9%	22.6%	38.4%	61	48.0%	39.5%	56.7%	26	20.5%	14.4%	28.3%	0	0.0%	0.0%	2.9%
6	Lezgins	123	7	5.7%	2.8%	11.3%	37	30.1%	22.7%	38.7%	62	50.4%	41.7%	59.1%	17	13.8%	8.8%	21.0%	0	0.0%	0.0%	3.0%
7	Kabardians	203	7	3.4%	1.7%	6.9%	46	22.7%	17.4%	28.9%	119	58.6%	51.7%	65.2%	27	13.3%	9.3%	18.7%	4	2.0%	0.8%	5.0%
8	Balkars	196	0	0.0%	0.0%	1.9%	51	26.0%	20.4%	32.6%	125	63.8%	56.8%	70.2%	14	7.1%	4.3%	11.6%	6	3.1%	1.4%	6.5%
9	Ossetians	244	4	1.6%	0.6%	4.1%	54	22.1%	17.4%	27.7%	118	48.4%	42.2%	54.6%	65	26.6%	21.5%	32.5%	3	1.2%	0.4%	3.6%
10	Tatars	141	11	7.8%	4.4%	13.4%	36	25.5%	19.1%	33.3%	53	37.6%	30.0%	45.8%	36	25.5%	19.1%	33.3%	5	3.5%	1.5%	8.0%
11	Mari	206	18	8.7%	5.6%	13.4%	69	33.5%	27.4%	40.2%	69	33.5%	27.4%	40.2%	47	22.8%	17.6%	29.0%	3	1.5%	0.5%	4.2%
12	Chuvash	236	16	6.8%	4.2%	10.7%	86	36.4%	30.6%	42.8%	94	39.8%	33.8%	46.2%	40	16.9%	12.7%	22.3%	0	0.0%	0.0%	1.6%
13	Mordvin-Moksha	109	3	2.8%	0.9%	7.8%	25	22.9%	16.0%	31.7%	52	47.7%	38.6%	57.0%	25	22.9%	16.0%	31.7%	4	3.7%	1.4%	9.1%
14	Buryats	114	1	0.9%	0.2%	4.8%	11	9.6%	5.5%	16.5%	54	47.4%	38.4%	56.5%	45	39.5%	31.0%	48.6%	3	2.6%	0.9%	7.5%
15	Nanais	115	0	0.0%	0.0%	3.2%	2	1.7%	0.5%	6.1%	51	44.3%	35.6%	53.5%	53	46.1%	37.3%	55.2%	9	7.8%	4.2%	14.2%

Note: UM – Ultrarapid Metabolizer; RM – Rapid Metabolizer; NM – Normal Metabolizer; IM – Intermediate Metabolizer; PM – Poor Metabolizer (CYP2C19 phenotype categories assigned per CPIC diplotype–phenotype tables); n – number of individuals in the given phenotype category; % – phenotype frequency; CI lower, % / CI upper, % – lower and upper bounds of the 95% Wilson confidence interval.
