# Supplementary material for: From Frequencies to Priorities: Interethnic Distribution of Pharmacogenetic Phenotypes as a Basis for Prioritizing Preemptive Testing in Russia’s Multiethnic Population
Source: Genes (Basel). 2026 Oct 14;17(10):1275. doi: 10.3390/genes17101275 (PMC13704235; doi:10.3390/genes17101275)
Supplement: Supplementary file 1 [file genes-17-01275-s001.zip › Table S4.pdf]

Table S4: Distribution of CYP3A5 phenotypes (Normal, Intermediate, Poor Metabolizer) in 15 ethnic groups of Russia, with 95% CIs.

#	Ethnic groups	Sample size. n	Normal Metabolizer (NM)				Intermediate Metabolizer (IM)				Poor Metabolizer (PM)			
			n	%	CI lower, %	CI upper, %	n	%	CI lower, %	CI upper, %	n	%	CI lower, %	CI upper, %
1	Russians	136	2	1.5%	0.4%	5.2%	15	11.0%	6.8%	17.4%	119	87.5%	80.9%	92.0%
2	Avars	117	2	1.7%	0.5%	6.0%	6	5.1%	2.4%	10.7%	109	93.2%	87.1%	96.5%
3	Dargins	121	2	1.7%	0.5%	5.8%	8	6.6%	3.4%	12.5%	111	91.7%	85.5%	95.4%
4	Laks	116	1	0.9%	0.2%	4.7%	8	6.9%	3.5%	13.0%	107	92.2%	85.9%	95.9%
5	Kumyks	127	1	0.8%	0.1%	4.3%	10	7.9%	4.3%	13.9%	116	91.3%	85.2%	95.1%
6	Lezgins	123	2	1.6%	0.4%	5.7%	8	6.5%	3.3%	12.3%	113	91.9%	85.7%	95.5%
7	Kabardians	203	0	0.0%	0.0%	1.9%	25	12.3%	8.5%	17.5%	178	87.7%	82.5%	91.5%
8	Balkars	196	0	0.0%	0.0%	1.9%	29	14.8%	10.5%	20.4%	167	85.2%	79.6%	89.5%
9	Ossetians	244	1	0.4%	0.1%	2.3%	23	9.4%	6.4%	13.7%	220	90.2%	85.8%	93.3%
10	Tatars	141	1	0.7%	0.1%	3.9%	20	14.2%	9.4%	20.9%	120	85.1%	78.3%	90.0%
11	Mari	206	4	1.9%	0.8%	4.9%	45	21.8%	16.7%	28.0%	157	76.2%	70.0%	81.5%
12	Chuvash	236	4	1.7%	0.7%	4.3%	44	18.6%	14.2%	24.1%	188	79.7%	74.1%	84.3%
13	Mordvin-Moksha	109	1	0.9%	0.2%	5.0%	21	19.3%	13.0%	27.7%	87	79.8%	71.3%	86.3%
14	Buryats	114	1	0.9%	0.2%	4.8%	25	21.9%	15.3%	30.4%	88	77.2%	68.7%	83.9%
15	Nanais	115	2	1.7%	0.5%	6.1%	11	9.6%	5.4%	16.3%	102	88.7%	81.6%	93.3%

Note: NM - Normal Metabolizer; IM – Intermediate Metabolizer; PM – Poor Metabolizer (nonexpressor) (CYP3A5 phenotype categories assigned per CPIC diplotype–phenotype tables); n – number of individuals in the given phenotype category; % – phenotype frequency; CI lower, % / CI upper, % – lower and upper bounds of the 95% Wilson confidence interval.
