# Supplementary material for: From Frequencies to Priorities: Interethnic Distribution of Pharmacogenetic Phenotypes as a Basis for Prioritizing Preemptive Testing in Russia’s Multiethnic Population
Source: Genes (Basel). 2026 Oct 14;17(10):1275. doi: 10.3390/genes17101275 (PMC13704235; doi:10.3390/genes17101275)
Supplement: Supplementary file 1 [file genes-17-01275-s001.zip › Table S5.pdf]

Table S5: Distribution of SLCO1B1 phenotypes (Normal, Decreased, Poor Function) in 15 ethnic groups of Russia, with 95% CIs.

#	Ethnic groups	Sample size. n	Normal Function (c.521TT)				Decreased Function (c.521TC)				Poor Function (c.521CC)			
			n	%	CI lower, %	CI upper, %	n	%	CI lower, %	CI upper, %	n	%	CI lower, %	CI upper, %
1	Russians	136	86	63.2%	54.9%	70.9%	44	32.4%	25.1%	40.6%	6	4.4%	2.0%	9.3%
2	Avars	117	86	73.5%	64.9%	80.7%	31	26.5%	19.3%	35.1%	0	0.0%	0.0%	3.2%
3	Dargins	121	108	89.3%	82.5%	93.6%	12	9.9%	5.8%	16.5%	1	0.8%	0.1%	4.5%
4	Laks	116	99	85.3%	77.8%	90.6%	16	13.8%	8.7%	21.2%	1	0.9%	0.2%	4.7%
5	Kumyks	127	119	93.7%	88.1%	96.8%	8	6.3%	3.2%	11.9%	0	0.0%	0.0%	2.9%
6	Lezgins	123	101	82.1%	74.4%	87.9%	22	17.9%	12.1%	25.6%	0	0.0%	0.0%	3.0%
7	Kabardians	203	144	70.9%	64.3%	76.7%	58	28.6%	22.8%	35.1%	1	0.5%	0.1%	2.7%
8	Balkars	196	151	77.0%	70.7%	82.4%	43	21.9%	16.7%	28.2%	2	1.0%	0.3%	3.6%
9	Ossetians	244	219	89.8%	85.3%	93.0%	22	9.0%	6.0%	13.3%	3	1.2%	0.4%	3.6%
10	Tatars	141	96	68.1%	60.0%	75.2%	41	29.1%	22.2%	37.0%	4	2.8%	1.1%	7.1%
11	Mari	206	147	71.4%	64.8%	77.1%	50	24.3%	18.9%	30.6%	9	4.4%	2.3%	8.1%
12	Chuvash	236	166	70.3%	64.2%	75.8%	65	27.5%	22.2%	33.6%	5	2.1%	0.9%	4.9%
13	Mordvin-Moksha	109	77	70.6%	61.5%	78.4%	28	25.7%	18.4%	34.6%	4	3.7%	1.4%	9.1%
14	Buryats	114	78	68.4%	59.4%	76.2%	31	27.2%	19.9%	36.0%	5	4.4%	1.9%	9.9%
15	Nanais	111	81	73.0%	64.0%	80.4%	26	23.4%	16.5%	32.1%	4	3.6%	1.4%	8.9%

Note: Normal Function (c.521TT), Decreased Function (c.521TC), and Poor Function (c.521CC) – predicted SLCO1B1 transporter function categories assigned per the CPIC diplotype-phenotype table; n – number of individuals in the given category; % – category frequency; CI lower, % / CI upper, % – lower and upper bounds of the 95% Wilson confidence interval.
