# Supplementary material for: From Frequencies to Priorities: Interethnic Distribution of Pharmacogenetic Phenotypes as a Basis for Prioritizing Preemptive Testing in Russia’s Multiethnic Population
Source: Genes (Basel). 2026 Oct 14;17(10):1275. doi: 10.3390/genes17101275 (PMC13704235; doi:10.3390/genes17101275)
Supplement: Supplementary file 1 [file genes-17-01275-s001.zip › Table S6.pdf]

Table S6: Comparison of frequencies of clinically significant composite phenotype categories of CYP2C9, CYP2C19, CYP3A5, and SLCO1B1 between each of the 14 ethnic groups and the Russian reference group (2×2 Fisher's exact test, Benjamini-Hochberg corrected).

#	Ethnic groups	N	CYP2C9 IM+PM				CYP2C19 UM+RM				CYP2C19 IM+PM				CYP3A5 PM				SLCO1B1 Decreased+Poor Function			
			n	%	p-value	p_adj	n	%	p-value	p_adj	n	%	p-value	p_adj	n	%	p-value	p_adj	n	%	p-value	p_adj
1	Russians	136	47	34.56%	-	-	51	37.50%	-	-	39	28.68%	-	-	119	87.50%	-	-	50	36.76%	-	-
2	Avars	117	49	41.88%	0.244612	0.380508	33	28.21%	0.140970	0.246698	26	22.22%	0.252237	0.398353	109	93.16%	0.145011	0.406031	31	26.50%	0.104597	0.206131
3	Dargins	121	47	38.84%	0.517544	0.658693	41	33.88%	0.602697	0.649059	27	22.31%	0.256084	0.398353	111	91.74%	0.311932	0.507733	13	10.74%	1.09E-06	5.10E-06
4	Laks	116	55	47.41%	0.040520	0.113455	26	22.41%	0,0133	0,0464	39	33.62%	0,415	0.528	107	92.24%	0.298991	0.507733	17	14.66%	9.55E-05	0.000334
5	Kumyks	127	65	51.18%	0.008663	0.030322	40	31.50%	0.364027	0.509637	26	20.47%	0.152521	0.305042	116	91.34%	0.326399	0.507733	8	6.30%	8.46E-10	8.73E-09
6	Lezgins	123	80	65.04%	1.09E-06	7.61E-06	44	35.77%	0.797347	0.797347	17	13.82%	0.004074	0.014257	113	91.87%	0.310154	0.507733	22	17.89%	0.000822	0.002302
7	Kabardians	203	89	43.84%	0.091358	0.213169	53	26.11%	0.030570	0.071331	31	15.27%	0.003872	0.014257	178	87.68%	1.000000	1.000000	59	29.06%	0.154948	0.214139
8	Balkars	196	86	43.88%	0.110705	0.221409	51	26.02%	0.029621	0.071331	20	10.20%	1.96E-05	0.000274	167	85.20%	0.629166	0.734027	45	22.96%	0.006792	0.015848
9	Ossetians	244	86	35.25%	0.911150	0.911150	58	23.77%	0.006348	0.029623	68	27.87%	0.905490	0.975144	220	90.16%	0.490641	0.686897	25	10.25%	1.25E-09	8.73E-09
10	Tatars	141	45	31.91%	0.702251	0.781745	47	33.33%	0.530011	0.618346	41	29.08%	1.000000	1.000000	120	85.11%	0.603400	0.734027	45	31.91%	0.447890	0.447890
11	Mari	206	67	32.52%	0.725906	0.781745	87	42.23%	0.430846	0.548350	50	24.27%	0.380070	0.532098	157	76.21%	0.011438	0.160129	59	28.64%	0.124233	0.206131
12	Chuvash	236	73	30.93%	0.490961	0.658693	102	43.22%	0.324928	0.505444	40	16.95%	0.008706	0.024376	188	79.66%	0.065209	0.304309	70	29.66%	0.168252	0.214139
13	Mordvin-Moksha	109	29	26.61%	0.211611	0.370319	28	25.69%	0.054941	0.109881	29	26.61%	0.774841	0.903981	87	79.82%	0.115617	0.404658	32	29.36%	0.275796	0.321762
14	Buryats	114	8	7.02%	8.56E-08	1.20E-06	12	10.53%	8.77E-07	6.14E-06	48	42.11%	0.032852	0.076655	88	77.19%	0.042772	0.299403	36	31.58%	0.424113	0.447890
15	Nanais	115	16	13.91%	0.000225	0.001051	2	1.74%	8.46E-14	1.18E-12	62	53.91%	5.94E-05	0.000415	102	88.70%	0.846460	0.911572	30*	27.03%	0.132513	0.206131

Note: N – total sample size of the ethnic group; n – number of individuals in the merged clinically significant phenotype category; % – corresponding frequency; p-value – two-sided p-value from the 2×2 Fisher's exact test comparing the given ethnic group with the Russian reference group; p_adj – Benjamini–Hochberg false discovery rate–adjusted p-value.

CYP2C9 IM+PM – combined Intermediate + Poor Metabolizer proportion; CYP2C19 UM+RM – combined Ultrarapid + Rapid Metabolizer proportion; CYP2C19 IM+PM – combined Intermediate + Poor Metabolizer proportion; CYP3A5 PM - CYP3A5 Poor Metabolizer proportion; SLCO1B1 Decreased+Poor Function – combined Decreased + Poor Function proportion.

* - SLCO1B1 n = 111; four samples failed genotyping for c.521T>C.
